# Opioid prescribing trends in pain management clinics in Taiwan: 2008–2018: A population-based retrospective study

**DOI:** 10.1097/MD.0000000000049706

**Published:** 2026-07-10

**Authors:** Kwok-On Ng, Kun-Yu Kao, Chung-Han Ho, Chia-Hung Yu, Jhi-Joung Wang, Chin-Chen Chu

**Affiliations:** a Department of Anesthesiology, Ditmanson Medical Foundation Chia Yi Christian Hospital, Chia Yi, Taiwan; b Department of Anesthesiology, Chi Mei Medical Center, Tainan, Taiwan; c Department of Medical Research, Chi Mei Medical Center, Tainan, Taiwan; d Department of Information Management, Southern Taiwan University of Science and Technology, Tainan, Taiwan; e Department of Computer Science and Information Engineering, Southern Taiwan University of Science and Technology, Tainan, Taiwan.

**Keywords:** cancer pain, chronic pain, opioid prescriptions, pain clinics, Taiwan, trends

## Abstract

Pain management clinics (PMCs) play a central role in managing complex pain conditions, yet nationwide data on opioid prescription trends in this specialized setting remain limited. This study examined opioid prescribing patterns in Taiwanese PMCs from 2008 to 2018. This nationwide retrospective population-based study used data from the Taiwan National Health Insurance Research Database. Adult patients aged 18 years or older who received at least 1 opioid prescription during outpatient PMC visits between 2008 and 2018 were included. Opioid medications were identified using Anatomical Therapeutic Chemical classification codes. Prescription trends by opioid type, associated diagnoses, hospital level, and age group were analyzed using linear regression. A total of 2,37,766 opioid prescriptions were issued across PMCs during the study period. Annual prescription counts fluctuated without a significant overall trend (*P* = .49), and prescriptions per patient remained stable (range: 2.92–3.49; *P* = .87), indicating that volume changes reflected patient number fluctuations rather than escalating prescribing intensity. Morphine was the most prescribed opioid, while pethidine declined markedly; oxycodone, introduced in 2016, surged to 4024 prescriptions by 2018. The leading indications were chronic pancreatitis (23.98%), oral cancer (15.26%), and neuropathic pain (12.35%), with non-cancer pain comprising 65.8% to 71.3% of prescriptions annually. Prescription rates increased significantly in patients aged 51 to 64 years (*P* = .04), and medical centers showed a significant upward trend in prescribing per 100 beds (*P* < .001). Opioid prescribing in Taiwanese PMCs was stable overall, with a shift from traditional agents toward newer high-potency opioids. The predominance of non-cancer pain indications and the centralization of prescribing within medical centers highlight the need for targeted stewardship policies and post-market surveillance of newly introduced opioids.

## 1. Introduction

Pain-related chief complaints and diseases have a profound impact on individuals and society globally.^[1]^ The burden of pain spans acute conditions, malignancy-related discomfort, and long-term nonmalignant chronic pain, often impairing function and quality of life. Opioid-based medications remain among the most effective pharmacological treatments for moderate to severe acute, chronic, and cancer pain.^[2,3]^ Over the past decades, there has been a marked increase in opioid prescriptions worldwide, which has raised concerns regarding their overuse, adverse outcomes, and societal consequences.^[4,5]^

The increase in opioid prescriptions has been paralleled by the growing crisis of prescription drug misuse, overdose, and opioid use disorders. This presents a clinical dilemma: The imperative to effectively relieve pain must be balanced against the risks of addiction, misuse, and associated morbidities. Consequently, the Centers for Disease Control and Prevention (CDC) recommends that opioids should not be considered a first-line therapy for chronic pain and should only be prescribed when the anticipated benefits outweigh the potential risks of treatment.^[2,4]^

Pain management clinics (PMCs) are specialized facilities dedicated to the diagnosis and treatment of chronic pain.^[6]^ These clinics commonly adopt a multidisciplinary approach incorporating pharmacologic, physical, and psychological therapies to address complex pain syndromes.^[7]^ Due to the severity and refractoriness of conditions treated, PMCs are often a significant source of opioid prescriptions, and their providers contribute substantially to the overall volume of opioid prescribing.^[8,9]^

Despite the central role of PMCs in managing complex pain, there is a paucity of literature analyzing opioid prescription trends in this setting.^[10]^ The role of practitioner specialty in opioid prescribing practices remains largely unknown, with conflicting results across studies.^[11,12]^ To our knowledge, no previous nationwide study has examined the longitudinal patterns of opioid prescribing in pain clinics or identified specific opioid preparations driving usage.

This study used a nationally representative dataset from Taiwan’s National Health Insurance Research Database (NHIRD) to track the annual volume, type, and associated diagnoses of prescribed opioid medications in pain clinics between 2008 and 2018. We also analyzed changes over time and stratified patterns by hospital level to better understand the dynamics of opioid use in this specialized care environment.

## 2. Materials and methods

### 2.1. Data source

Taiwan implemented a single-payer National Health Insurance (NHI) program in 1995, covering over 99.99% of its population of approximately 23 million residents. This study utilized the NHIRD, which contains de-identifiedpatient-level claims data including outpatient, inpatient, and prescription records, along with demographic and diagnostic information. Diagnostic data were coded using the International Classification of Diseases, Ninth Revision, Clinical Modification (ICD-9-CM) prior to 2016, and the Tenth Revision (ICD-10-CM) thereafter.^[13]^ The NHIRD has been validated for epidemiological research and reflects nationwide healthcare utilization.^[14]^ Moreover, because all types of personal identification were encrypted to secure patient privacy, the current study was granted an exemption from a full ethical review by the Chi Mei Hospital Institutional Review Board (IRB-10812-E01).

### 2.2. Study design, patient identification, and variables

**Figure 1.**
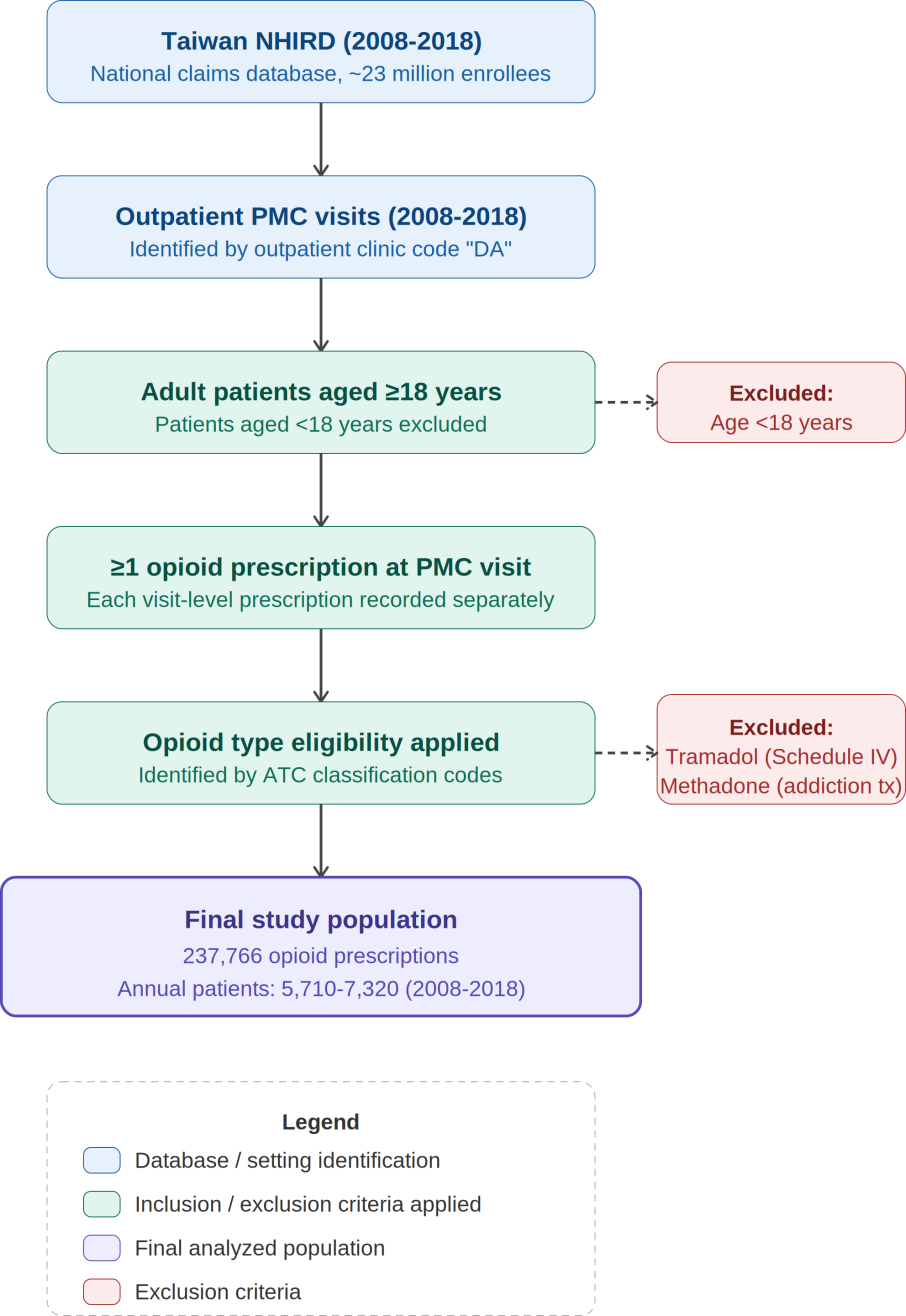
Flowchart illustrating the study population selection process from the Taiwan National Health Insurance Research Database (NHIRD) to the final analyzed population. ATC = Anatomical Therapeutic Chemical, NHIRD = National Health Insurance Research Database, PMC = pain management clinic.

This nationwide, retrospective, cross-sectional study included adult patients (≥18 years) visiting designated pain clinics in Taiwan from January 1, 2008, to December 31, 2018. This study utilized the outpatient claims database of the NHIRD. Since PMCs in Taiwan function predominantly as outpatient-based services, the outpatient database captures the majority of opioid prescribing activity within this clinical setting (Fig. 1 flow chart).

The study population included adult patients (aged ≥18 years) who visited a designated PMC (outpatient clinic code: DA) and received at least 1 opioid prescription between January 1, 2008, and December 31, 2018. Patients aged <18 years were excluded. Regarding opioid selection, tramadol was excluded owing to its classification as a weak mu-opioid receptor agonist and a Schedule IV controlled substance in Taiwan,^[15]^ and methadone was excluded as its use in Taiwan is restricted to opioid addiction treatment rather than pain management.^[16]^ The complete study population selection process is illustrated in Figure 1.

Opioid prescription events were recorded during the visit. In Taiwan’s NHI system, each opioid reimbursement claim requires a physician-issued narcotic prescription form; therefore, each claim record in the NHIRD corresponds to a discrete physician-initiated prescription event. Patients with multiple PMC visits during the study period were eligible for inclusion, with each visit-level opioid prescription recorded as a separate prescription event. The diagnosis associated with each prescription was extracted from the primary diagnosis field of the outpatient claim and subsequently categorized into major pain syndrome groups for analysis. Opioid medications were identified using Anatomical Therapeutic Chemical (ATC) classification codes; the complete list of ATC codes used for each opioid agent is provided in the Appendix Table, Supplemental Digital Content 1. Diagnoses were identified using ICD-9-CM codes for visits prior to 2016 and ICD-10-CM codes thereafter.^[13]^ Participating institutions were classified into 3 levels according to Taiwan’s NHI hospital accreditation system: medical centers, regional hospitals, and local hospitals (including clinics).

### 2.3. Study endpoints

The primary endpoint was the temporal trend of the annual volume of opioid prescriptions issued during outpatient PMC visits between 2008 and 2018, expressed both as total prescription counts and as prescriptions per patient per year. The secondary endpoints included: the distribution of opioid types identified by ATC classification codes; diagnoses associated with opioid prescriptions, captured from the primary ICD-9-CM/ICD-10-CM diagnosis field; and prescription volumes stratified by hospital level (medical centers, regional hospitals, and local hospitals) per 100 beds.

### 2.4. Statistical analysis

We conducted descriptive analyses using 9.4 (SAS Institute, Inc., Cary). To evaluate the temporal trends in opioid prescriptions from 2008 to 2018, linear regression analyses were performed with the annual number of prescriptions as the dependent variable and the calendar year as the independent variable. In addition to the overall trend, subgroup analyses were used to assess differences in annual prescription trends across hospital levels, top 9 disease categories, and different age groups. Trend differences between groups were assessed by including the interaction terms between calendar years and each subgroup variable in the regression models. Statistical significance was set at a *P*-value < .05.

## 3. Results

### 3.1. Trend of opioid prescription in nation and pain management clinics in Taiwan, 2008–2018

A total of 2,37,766 opioid prescriptions were identified in PMCs during the study period. As shown in Table 1, the annual number of patients receiving opioid prescriptions in PMCs ranged from 5710 to 7320. Despite fluctuations in total prescription counts, prescriptions per patient remained relatively stable, ranging from 2.92 to 3.49, with no significant temporal trend (*P* = .87). Similarly, total prescription counts in PMCs did not show a significant trend over time (*P* = .49).

**Table 1:** Trends in opioid prescriptions at the national level and in pain management clinics (PMCs) in Taiwan, 2008–2018.

Year	2008	2009	2010	2011	2012	2013	2014	2015	2016	2017	2018
National level											
Total prescriptions	16,57,871	16,94,034	17,16,238	17,53,601	17,91,098	17,58,148	18,38,697	18,59,456	18,90,447	19,45,930	18,84,408
Number of patients	6,30,661	6,53,020	6,56,337	6,59,402	6,66,428	6,48,452	6,71,991	6,77,342	6,85,489	6,82,429	6,78,124
Prescriptions per patient	2.63	2.59	2.61	2.66	2.69	2.71	2.74	2.75	2.76	2.85	2.78
Pain management clinics (PMCs)											
Total prescriptions	20,362	20,785	21,781	22,854	23,404	23,875	24,255	20,831	20,017	18,426	21,176
Number of patients	6964	6693	6244	6687	6851	7320	7293	6150	6018	5710	7014
Prescriptions per patient	2.92	3.11	3.49	3.42	3.42	3.26	3.33	3.39	3.33	3.23	3.02
PMC prescriptions (% of national total)	1.23	1.23	1.27	1.30	1.31	1.36	1.32	1.12	1.06	0.95	1.12

Data were derived from the NHIRD and represent reimbursed opioid prescriptions. “Prescriptions per patient” was calculated as the annual number of prescriptions divided by the number of patients receiving at least 1 opioid prescription. PMC (%) indicates the proportion of prescriptions relative to the national total. Tramadol and methadone was excluded. No significant temporal trend was observed in PMC prescriptions (*P* = .49) or prescriptions per patient (*P* = .87).

NHIRD = National Health Insurance Research Database, PMC = pain management clinic.

At the national level, the number of patients varied modestly over time from 6,30,661 in 2008 to 6,78,124 in 2018, accompanied by a gradual increase in prescriptions per patient from 2.63 to 2.78. In contrast, PMC prescriptions consistently accounted for a small proportion of total national opioid prescriptions, ranging from 0.95% to 1.36% during the study period.

### 3.2. Trends in cancer and non-cancer opioid prescriptions in pain management clinics, 2008–2018

**Figure 2.**
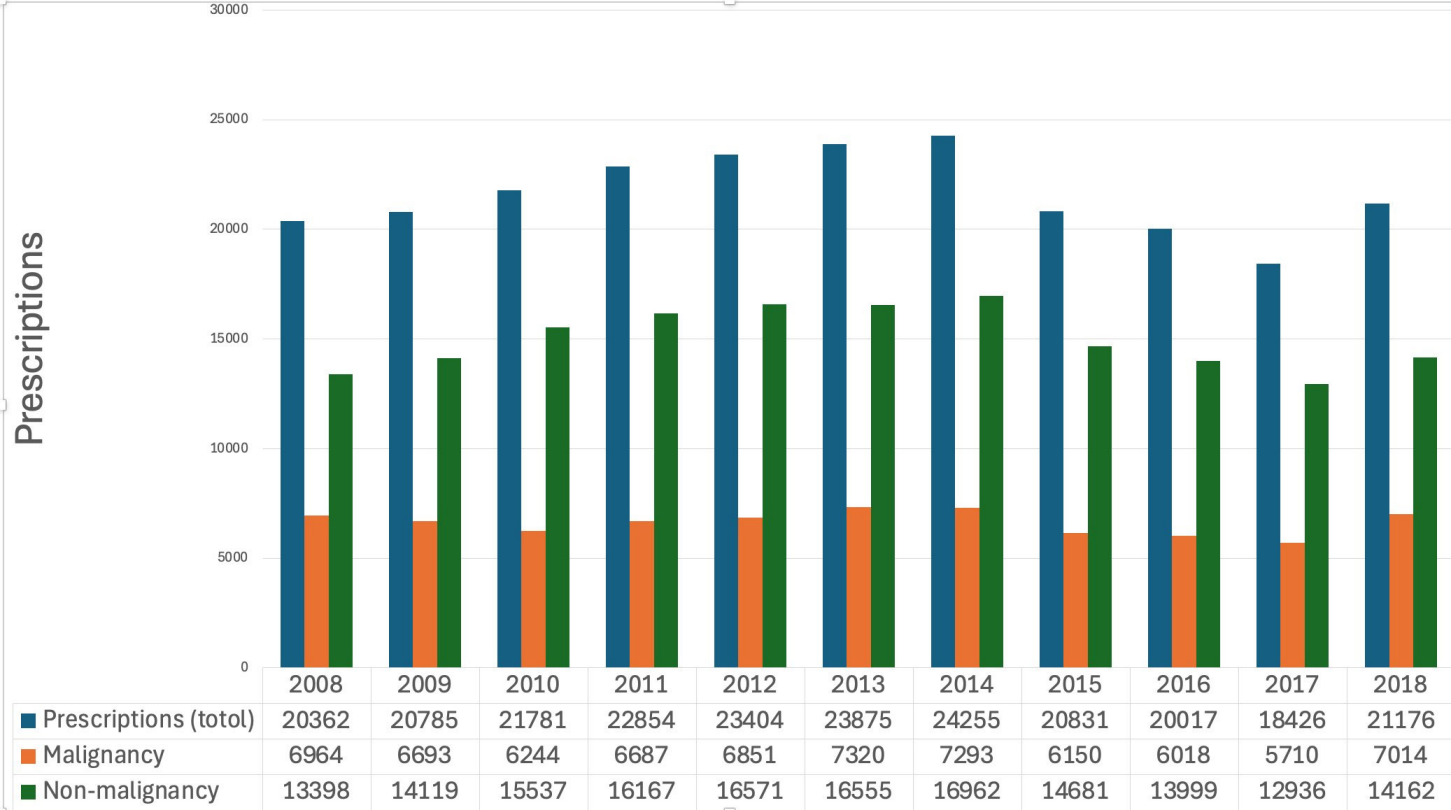
Opioid prescription in pain management clinics, stratified by cancer versus non-cancer, 2008–2018 in Taiwan.

Between 2008 and 2018, 20,362 to 24,255 opioid prescriptions were issued annually by PMCs. The proportion of prescriptions for cancer-related pain steadily decreased from 34.2% in 2008 to 28.67% in 2010 with minor annual fluctuations. By 2018, the proportion of cancer-related prescriptions had increased slightly to 33.12%. In absolute numbers, cancer prescriptions ranged from 5710 to 7320 annually (Fig. 2).

Conversely, prescriptions for non-cancer-related pain consistently comprised most total PMC opioid prescriptions throughout the study period. Non-cancer indications accounted for 65.80% of prescriptions in 2008, peaking at 71.33% in 2010, and subsequently fluctuating between 66.88% and 70.83% in later years. The absolute number of non-cancer prescriptions ranged from 12,936 to 16,962.

### 3.3. *Trends in opioid prescriptions by drug type* (Fig. 3)

Between 2008 and 2018, the total number of opioid prescriptions issued in PMCs in Taiwan varied from 18,426 to 24,255 annually, peaking in 2014. Morphine consistently accounted for the largest proportion of prescriptions throughout the study period, rising from 12,587 in 2008 to a peak of 14,956 in 2014, followed by a slight decline and stabilization around 12,801 in 2018. Pethidine prescriptions exhibited a marked and continuous decline, dropping from 2672 in 2008 to 1187 in 2018. Fentanyl prescriptions increased gradually from 3452 in 2008 to 4822 in 2018. Codeine use remained low throughout the study period, decreasing from 564 to 257 prescriptions. Prescriptions for buprenorphine showed a gradual upward trend. Notably, the introduction of newer opioids caused significant changes in prescription patterns; hydromorphone, introduced in 2014, and oxycodone, introduced in 2016, both exhibited rapid increases in utilization immediately after market entry, with oxycodone surging from negligible use before 2016 to 4024 prescriptions by 2018. These trends collectively reflect a gradual shift from traditional opioids, such as pethidine and codeine, toward more potent or longer-acting agents, including fentanyl, hydromorphone, and oxycodone.

**Figure 3.**
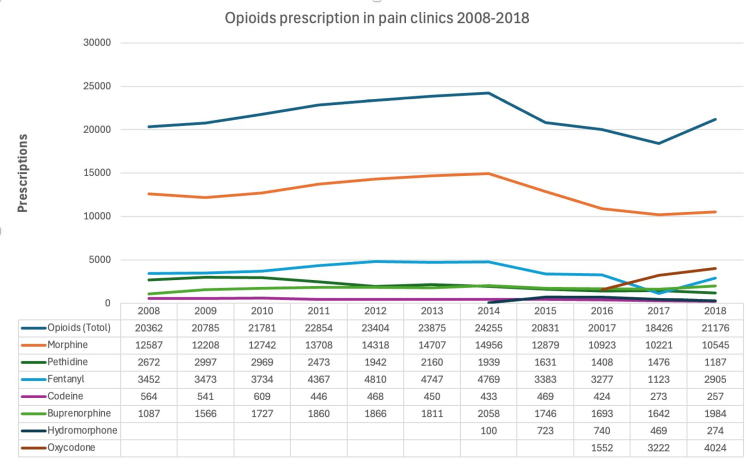
Trends in opioid prescriptions in pain management clinics in Taiwan, 2008–2018, classified by specific opioid agent. Trend analysis by linear regression: morphine (*P* = .26), fentanyl (*P* < .01, increased), pethidine (*P* < .01, decreased), codeine (*P* < .01, decreased), buprenorphine (*P* = .08), hydromorphone (*P* = .11), and oxycodone (*P* = .06).

### 3.4. *Diagnoses associated with opioid prescriptions* (Figs. 4 and 5)

From 2008 to 2018, the most common indications for opioid prescriptions in adult PMCs were chronic pancreatitis (9528 prescriptions, 23.98%), oral cancer (6064; 15.26%), and neuropathic pain (4909; 12.35%). Other frequent diagnoses include post-laminectomy syndrome (3923), lung cancer (3770), myofascitis or myositis (3200), colorectal and anal cancer (3163), reflex sympathetic dystrophy (2689), and lumbosacral spine degeneration (2493). These conditions accounted for the majority of opioid prescriptions during the study period. Notably, an increasing trend was observed in prescriptions related to neuropathic and musculoskeletal pain, underscoring the significant role of both cancer-related and chronic non-cancer pain (CNCP) in opioid use in Taiwanese pain clinics.

**Figure 4.**
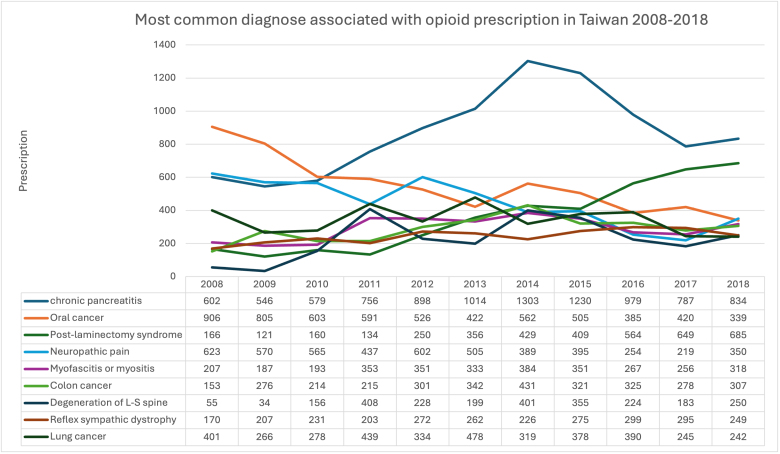
Annual trends in opioid prescriptions for the 9 most common diagnoses in pain management clinics in Taiwan, 2008–2018. Trend analysis: oral cancer (*P* < .01, decreased), neuropathic pain (*P* < .01, decreased), PLS (*P* < .01, increased), RSD (*P* < .01, increased). PLS = post-laminectomy syndrome; RSD = reflex sympathetic dystrophy.

**Figure 5.**
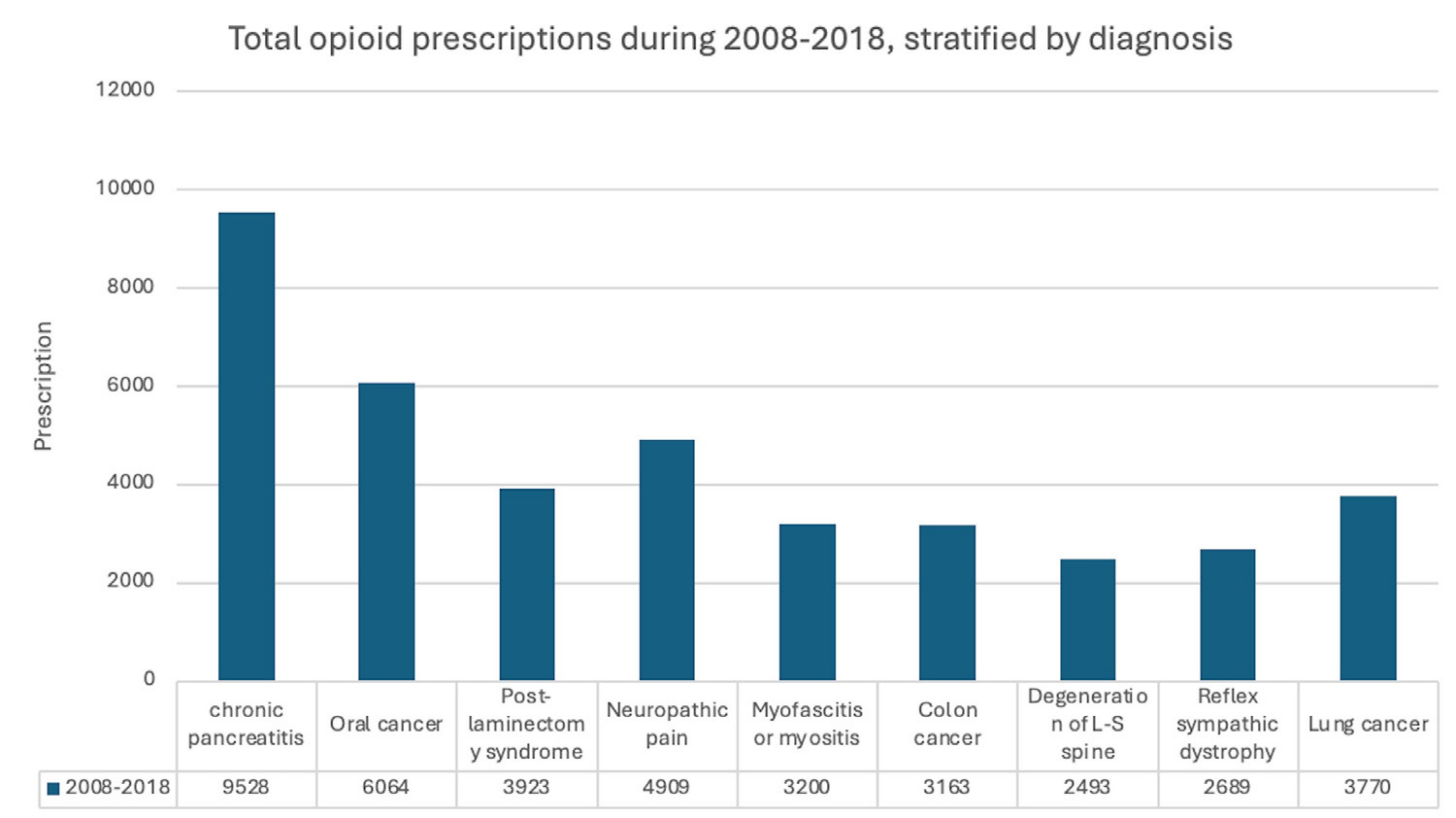
Total cumulative opioid prescriptions by primary diagnosis in pain management clinics in Taiwan, 2008–2018.

### 3.5. *Age-specific trends in opioid prescription rates (2008–2018;* Fig. 6)

**Figure 6.**
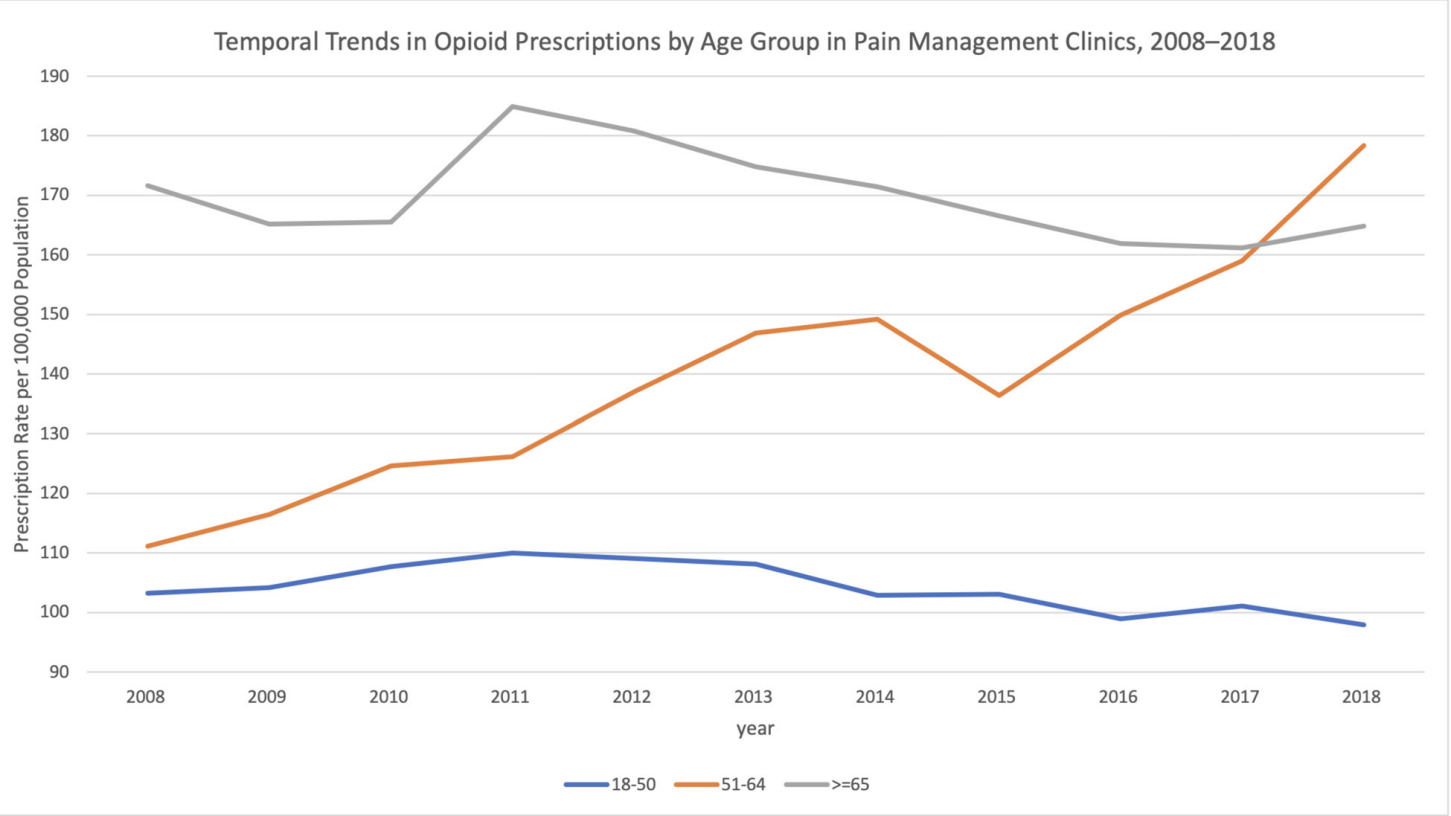
Temporal trends in age-specific opioid prescription rates per 1,00,000 population in pain management clinics in Taiwan, 2008–2018, stratified by age group (18–50, 51–64, and ≥65 years).

Figure 6 presents the temporal trends in age-specific opioid prescription rates per 10^5^ individuals from 2008 to 2018. From 2008 to 2018, the age-specific prescription rates per 10^5^ population demonstrated varying trends between age groups (*P* < .001). The 51 to 64 years age group exhibited a statistically significant upward trend, with the prescription rate increasing from 111/10^5^ in 2008 to 178/10^5^ in 2018 (*P* = .04). For the 18 to 50-year age group, the prescription rate also presented a slight decreasing trend from 103/10^5^ in 2008 to 98/10^5^ in 2018 (*P* < .001). For individuals aged ≥65 years, the prescription rate initially increased to a peak in 2011 to 2012 but declined thereafter, resulting in an overall flat trend without statistical significance (*P* = .20).

### 3.6. *Hospital-level trends in opioid prescriptions* (Fig. 7)

**Figure 7.**
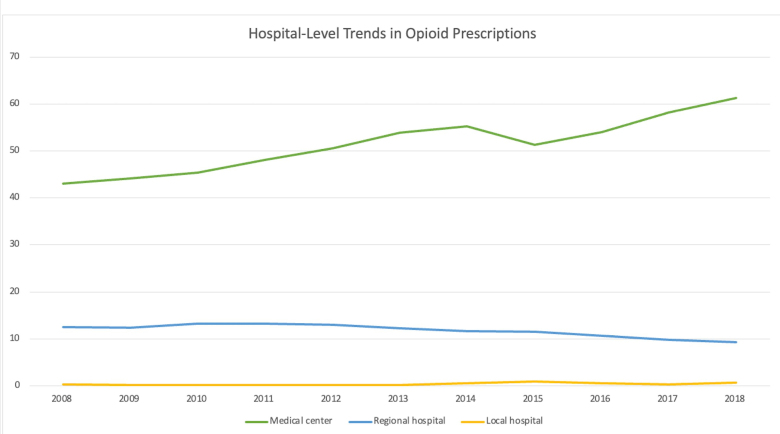
Trends in opioid prescriptions per 100 hospital beds in pain management clinics in Taiwan, 2008–2018, stratified by hospital accreditation level (medical centers, regional hospitals, and local hospitals including clinics).

To evaluate the differences in opioid prescription patterns across hospital levels, we analyzed the total number of opioid prescriptions standardized by 100 hospital beds from 2008 to 2018. As shown in Figure 7, medical centers consistently prescribed the highest volume of opioids per 100 beds, followed by regional and local hospitals. Over the 11-year period, opioid prescriptions in medical centers showed a statistically significant increasing trend (*P* < .001). In contrast, regional hospitals exhibited a significant downward trend (*P* < .001), and local hospitals maintained a stable but minimal prescription volume (*P* = .04). These findings indicate that, based on trend analysis, tertiary-level institutions have played a predominant role in opioid dispensing within PMCs in Taiwan, with significant differences observed across the 3 healthcare levels (*P* < .001).

## 4. Discussion

### 4.1. Summary of key findings

This nationwide study examined opioid prescribing trends in Taiwanese PMCs over an 11-year period. Although absolute prescription volumes fluctuated from year to year, prescriptions per patient remained stable throughout the study period (2.92–3.49; *P* = .87), indicating that changes in total prescription counts were driven primarily by fluctuations in the number of patients receiving care rather than by escalating prescribing intensity. Morphine and fentanyl remained the most commonly prescribed opioids, pethidine use declined markedly, and newer agents such as hydromorphone and oxycodone gained rapid uptake following their market introduction. Medical centers accounted for a disproportionate share of prescribing volume relative to other hospital levels, and non-cancer pain consistently accounted for the majority of indications. These findings are discussed below in relation to the national prescribing landscape, the international literature, diagnosis-specific patterns, age-related trends, and their implications for opioid stewardship in Taiwan.

### 4.2. PMC prescribing in the national and cross-setting context

Nationally, total opioid prescriptions in Taiwan increased gradually between 2008 and 2018, with morphine as the most commonly prescribed agent and pethidine use declining substantially.^[17]^ Within this broader landscape, PMC-attributed prescriptions consistently represented a small share of the national total, ranging from 0.95% to 1.36% across the study period. For context, our previous analysis of emergency department (ED) opioid prescribing in Taiwan found that EDs accounted for 9.72% to 11.97% of the national total over a comparable period, roughly 8 to 10 times the PMC share.^[18]^ A direct within-dataset comparison of prescribing behavior between pain physicians and other specialists was beyond the scope of the present study, and this disparity should not be interpreted as a direct measure of physician-level prescribing intensity. It more plausibly reflects the differing clinical mandates of the 2 settings: PMCs manage long-term, often refractory pain requiring sustained opioid stewardship, while EDs address acute, episodic pain where short-course opioid use predominates. The relatively stable prescriptions-per-patient metric observed in PMCs is consistent with, though does not directly confirm, a more standardized prescribing approach within this specialized setting.

### 4.3. International context

Opioid prescribing in Taiwanese PMCs is best interpreted against the backdrop of substantial international heterogeneity. Across high-income countries, the prevalence of opioid prescribing for chronic pain varies widely, from as low as 2.2% in Japan to as high as 18.1% in France, with patients in many Asian healthcare systems consistently less likely to receive opioid therapy than their North American counterparts.^[19]^ Within this global pattern, our findings align most closely with other Asian and European health systems rather than with the United States. In South Korea, a survey of pain physicians across 42 university hospital pain clinics found a similarly cautious, opioid-conservative prescribing culture, with most physicians expressing reluctance to prescribe opioids for CNCP and citing concerns about addiction and regulatory scrutiny.^[12]^ In the United Kingdom, non-cancer pain has been reported to account for approximately 87.8% of strong opioid prescriptions in primary care,^[20]^ a proportion strikingly similar to the 65.8% to 71.3% observed in our PMC cohort, suggesting that the predominance of non-cancer indications for opioid prescribing is a pattern shared across diverse healthcare systems rather than a feature unique to Taiwan.

A 4-country population-based cohort study directly comparing opioid initiation for non-cancer pain across Taiwan, the United States, Canada, and the United Kingdom found that Taiwan had the lowest median starting dose (8 morphine milligram equivalents [MME]/d), compared with 12 in the UK, 23 to 27 in Canada, and 38 in the United States; oxycodone was the dominant first-line opioid in 65% of US patients but was virtually absent from Taiwanese prescribing.^[21]^ The contrast with the United States is particularly instructive, though it should be read as a cautionary benchmark rather than a direct parallel: the US opioid-related overdose mortality rate increased roughly fourfold between 1999 and 2010, driven by a combination of aggressive pharmaceutical marketing, a fragmented insurance system, and, for a period, permissive prescribing regulations, factors that have no close analogue within Taiwan’s single-payer, tightly regulated NHI system. Taken together, these international comparisons suggest that Taiwan’s PMC prescribing environment, characterized by low starting doses, minimal oxycodone use prior to 2016, and a predominance of non-cancer indications consistent with other Asian and European systems, is more analogous to conservative prescribing cultures elsewhere in Asia and Europe than to the trajectory historically observed in the United States.

### 4.4. Factors underlying conservative prescribing in Taiwan

Survey data from Taiwan offer some insight into the factors that may underlie this conservative prescribing pattern, although these explanations remain inferential rather than directly tested by the present dataset. Pain specialists in Taiwan have been reported to prescribe opioids more consistently, with less arbitrary dose or duration reduction, than non-pain physicians, and to demonstrate greater familiarity with narcotic regulations and chronic pain management principles.^[22]^ Conversely, non-pain physicians in Taiwan more frequently report reluctance to prescribe opioids and gaps in relevant training, citing concerns about misuse (reported by 78–92% of surveyed physicians) and inadequate knowledge of pain management (73–84%).^[22]^ If this pattern extends to the PMC setting, it would suggest that the relatively small share of national opioid prescribing attributable to PMCs may partly reflect broader opioid prescribing caution among non-pain physicians outside specialized clinics, rather than under-prescribing within PMCs themselves; we did not, however, directly test this relationship in our data, and it should be regarded as a plausible explanation rather than a confirmed finding. International medical graduates trained in the United States and Canada have also been shown to prescribe significantly higher opioid doses than those trained elsewhere, lending further support to the broader observation that prescribing norms are shaped substantially by national training and regulatory culture.^[23]^

### 4.5. Diagnosis-specific patterns

The diagnostic profile associated with opioid prescriptions in PMCs differed markedly from that observed in our earlier study of ED prescribing, where opioids were used predominantly for acute conditions such as limb fractures, abdominal pain, and renal colic.^[18]^ In PMCs, prescriptions were instead concentrated among chronic, often refractory conditions: chronic pancreatitis (23.98%), neuropathic pain (12.35%), post-laminectomy syndrome (9.87%), and degenerative musculoskeletal disease, alongside a substantial cancer-related burden from oral, lung, and colorectal malignancy. This contrast reflects the distinct clinical roles of the 2 settings: PMCs manage long-term, frequently refractory pain syndromes, while EDs address acute, episodic presentations.

The prominence of chronic pancreatitis and oral cancer as leading indications is consistent with the epidemiological burden of these conditions in Taiwan. Taiwan has one of the highest reported incidences of oral cancer worldwide, attributable in large part to the prevalence of betel nut chewing, particularly among middle-aged men, a pattern that aligns with our age-specific findings.^[24,25]^ For chronic pancreatitis, opioid therapy remains a guideline-endorsed mainstay for refractory pancreatic pain, including in the American College of Gastroenterology (ACG)’s clinical guidelines.^[26]^ These diagnosis-specific patterns therefore appear to reflect Taiwan’s underlying disease epidemiology more than any unique feature of PMC prescribing behavior.

Beyond individual diagnoses, the overall balance between cancer and non-cancer indications also merits attention, given that opioid use differs in pathophysiological rationale, treatment goals, and applicable guidelines between these 2 contexts.^[11]^ Non-cancer pain accounted for the majority of PMC opioid prescriptions throughout the study period (65.80–71.33% annually), while cancer-related prescriptions declined from 34.2% in 2008 to 28.67% in 2010 before a modest rebound to 33.12% by 2018; in Taiwan, most cancer pain is managed by oncology services rather than PMCs, which likely contributes to this distribution. This pattern is broadly consistent with international trends toward more cautious opioid use across both cancer and non-cancer populations: a large US community health center network reported a 73.7% decline in opioid prescriptions per 100 patients between 2009 and 2018,^[27]^ and an outpatient palliative care clinic documented a fivefold decline in median opioid dose and a >50% reduction in long-acting opioid prescriptions for cancer pain between 2016 and 2021.^[28]^

### 4.6. Age-specific trends

Opioid prescription rates rose significantly among adults aged 51 to 64 years over the study period (*P* = .04), while rates among adults aged 18 to 50 declined slightly, and rates among adults aged 65 years or older showed an early peak followed by a plateau without an overall significant trend. Several explanations are plausible, although none can be directly confirmed with the data available in this study. The 51 to 64 age group experiences a convergence of rising cancer incidence, particularly oral and lung cancer in Taiwan,^[29,30]^ together with an accumulating burden of CNCP conditions such as neuropathic pain and degenerative spinal disease. Unlike older patients, this age group may not yet carry the functional decline or comorbidity burden that often prompts more conservative opioid prescribing, and continued occupational activity in this group may also increase the clinical priority placed on functional pain control, though we did not have data on employment status to test this directly. The comparatively stable or declining rates among patients 65 years and older are consistent with, though not directly explained by, heightened clinical caution regarding opioid-related risks such as falls, cognitive impairment, and respiratory depression in older adults, a pattern reflected in geriatric prescribing guidelines that favor conservative opioid use in this population. These age-related patterns warrant continued surveillance, particularly given the growing burden of both cancer and non-cancer pain among middle-aged adults in Taiwan, though we emphasize that the explanations offered here are interpretive rather than data-confirmed.

### 4.7. Implications for opioid stewardship

Taiwan currently regulates strong opioids as Schedule II controlled substances under the Controlled Drugs Act, requiring specialized narcotic prescription forms and mandatory reporting to the Taiwan Food and Drug Administration (TFDA)’s Controlled Drug Management Information System (CDMIS).^[17]^ This regulatory framework provides an important foundation, but our findings point to several areas where it could be strengthened. The rapid increase in oxycodone prescriptions following its 2016 introduction points to the value of mandatory post-market prescribing audits for newly introduced high-potency opioids. The increasing concentration of prescribing within medical centers supports the development of structured inter-hospital referral protocols to help ensure equitable access to pain management across hospital levels. The sustained predominance of non-cancer pain as an indication, particularly among middle-aged adults, points to a need for differentiated stewardship protocols that distinguish cancer from non-cancer pain management and incorporate periodic review for long-term opioid users. Finally, given documented gaps in opioid-related knowledge and greater prescribing reluctance among non-pain physicians in Taiwan,^[22]^ structured continuing medical education targeting this group may help ensure more consistent, guideline-concordant pain management beyond the specialized PMC setting.

## 5. Limitations

This study has several limitations. First, the data reflect only the number of prescriptions, not the dosage, duration, or specific formulation, which may affect the interpretation of the opioid use burden. Second, the study did not differentiate between new and repeated prescriptions, thus limiting insights into patterns of chronic opioid use. Third, we could not confirm the appropriateness of each prescription or assess the clinical outcomes or the effectiveness of pain relief. Fourth, diagnostic accuracy depends on coding by healthcare providers, which may introduce a misclassification bias. Fifth, this study was based on the outpatient claims database; inpatient opioid prescriptions were not included. However, given that PMCs in Taiwan operate almost exclusively on an outpatient basis, this is unlikely to substantially affect the overall findings. Sixth, data on patient characteristics such as pain severity, functional impairment, or opioid-related adverse events were not available. Seventh, this study excluded tramadol due to its distinct pharmacological profile and its Schedule IV classification in Taiwan, which carries different prescription requirements from the Schedule II opioids analyzed in this study; this may underestimate the total opioid burden in PMCs. Finally, the database lacked provider-specific information; therefore, we could not assess prescribing behavior according to specialty or experience.

## 6. Conclusions

This nationwide analysis showed that opioid prescription volumes in PMCs fluctuated over time without a significant overall trend, and the relatively stable prescriptions-per-patient metric indicates that fluctuations in total prescription counts were driven primarily by changes in patient volume rather than escalating prescribing intensity. The sharp decline in pethidine use aligns with international safety trends, while the rapid uptake of newly introduced opioids such as oxycodone and hydromorphone warrants continued post-market surveillance. Medical centers were the predominant prescribers of opioids, and the leading indications of chronic pancreatitis and cancer-related pain syndromes were consistent with established international treatment guidelines, suggesting that opioid use in Taiwanese PMCs largely reflects guideline-concordant clinical practice. The growing burden of non-cancer pain indications, particularly in the middle-aged population, warrants continued surveillance and targeted stewardship policies that address the specific risks associated with high-potency opioids and the centralization of opioid prescribing within tertiary care settings. Taiwan’s experience, characterized by a single-payer NHI system, strict regulatory oversight, and a conservatively managed specialist-led prescribing environment, offers valuable insights for other Asian healthcare systems navigating the challenge of ensuring appropriate opioid access while preventing overuse.

## Acknowledgments

We thank the Health and Welfare Data Science Center and National Health Insurance Administration for providing the data used in this study.

## Author contributions

**Conceptualization:** Kwok-On Ng, Jhi-Joung Wang, Chin-Chen Chu.

**Data curation:** Kwok-On Ng, Chung-Han Ho, Chia-Hung Yu, Chin-Chen Chu.

**Formal analysis:** Chung-Han Ho, Chin-Chen Chu.

**Funding acquisition:** Jhi-Joung Wang.

**Investigation:** Kwok-On Ng, Jhi-Joung Wang, Chin-Chen Chu.

**Methodology:** Kwok-On Ng, Kun-Yu Kao, Chung-Han Ho, Jhi-Joung Wang, Chin-Chen Chu.

**Project administration:** Kun-Yu Kao, Chia-Hung Yu, Chin-Chen Chu.

**Software:** Kun-Yu Kao.

**Supervision:** Jhi-Joung Wang, Chin-Chen Chu.

**Validation:** Chia-Hung Yu, Chin-Chen Chu.

**Visualization:** Kwok-On Ng, Chia-Hung Yu, Chin-Chen Chu.

**Writing – original draft:** Kwok-On Ng, Kun-Yu Kao, Chung-Han Ho.

**Writing – review & editing:** Chin-Chen Chu.


