# Supplementary material for: Opioid prescribing trends in pain management clinics in Taiwan: 2008–2018: A population-based retrospective study
Source: Medicine (Baltimore). 2026 Jul 10;105(28):e49706. doi: 10.1097/MD.0000000000049706 (PMC13362942; doi:10.1097/MD.0000000000049706)
Supplement: Supplementary file 1 [file medi-105-e49706-s001.docx]

**Appendix Table. Anatomical Therapeutic Chemical (ATC) Codes for Opioid Medications Included in the Analysis**

| **Drug Category** | **Drug Name** | **ATC Code(s)** |
| --- | --- | --- |
| **Opioid** | Opioid (overall) | N02A* |
|  | Fentanyl | N01AH01, N02AB03, N01AH51 |
|  | Morphine | N02AA01, N02AG01, A07DA52, N02AA51, R05DA05 |
|  | Hydromorphone | N02AA03, N02AG04, N02AA53 |
|  | Oxycodone | N02AA05, N02AJ18, N02AJ19, N02AA55, N02AA56, N02AJ17 |
|  | Pethidine | N02AB02, N02AG03, N02AB52, N02AB72 |
|  | Buprenorphine | N02AE01, N07BC01, N07BC51 |
|  | Codeine | R05DA04, N02AJ07, N02AJ08, N02AJ09, N02AJ06, N02AA59, N02AA79 |

** N02A: Opioids (ATC class). All subclasses included unless otherwise specified. ATC = Anatomical Therapeutic Chemical classification system (WHO).*
